# The Time Course of the Influence of Valence and Arousal on the Implicit Processing of Affective Pictures

**DOI:** 10.1371/journal.pone.0029668

**Published:** 2012-01-25

**Authors:** Chunliang Feng, Lili Wang, Chao Liu, Xiangru Zhu, Ruina Dai, Xiaoqin Mai, Yue-Jia Luo

**Affiliations:** 1 State Key Laboratory of Cognitive Neuroscience and Learning, Beijing Normal University, Beijing, China; 2 Department of Psychology, Henan University, Henan, China; 3 Center for Human Growth and Development, University of Michigan, Ann Arbor, Michigan, United States of America; Cuban Neuroscience Center, Cuba

## Abstract

In the current study, we investigated the time course of the implicit processing of affective pictures with an orthogonal design of valence (negative *vs.* positive) by arousal (low *vs.* high). Previous studies with explicit tasks suggested that valence mainly modulates early event-related potential (ERP) components, whereas arousal mainly modulates late components. However, in this study with an implicit task, we observed significant interactions between valence and arousal at both early and late stages over both parietal and frontal sites, which were reflected by three different ERP components: P2a (100–200 ms), N2 (200–300 ms), and P3 (300–400 ms). Furthermore, there was also a significant main effect of arousal on P2b (200–300 ms) over parieto-occipital sites. Our results suggest that valence and arousal effects on implicit affective processing are more complicated than previous ERP studies with explicit tasks have revealed.

## Introduction

As one of the fundamental aspects of the human mind, emotion and its brain mechanisms have received increasing interest recently, especially with the development of non-invasive neuroimaging techniques such as functional magnetic resonance imaging (fMRI) and event-related potentials (ERPs) [1]. Compared to fMRI, the ERP technique can provide fine-grain temporal resolution of mental processing and thus has been widely used in studying the time course of affective processing. Studies have revealed that affective modulations can occur as early as around 100 ms after stimulus onset and can be sustained for as long as several seconds [2]. According to the dimensional view of emotion, affective valence (ranging from negative to positive) and arousal (ranging from calm to excited) are two primary dimensions of emotion [3]. A rich body of literature has suggested that these two dimensions modulate ERP components at different temporal stages with valence mainly affecting ERP components at an early stage (e.g., before 200 ms) and arousal mainly affecting ERP components at a later stage (e.g., after 250 ms) [2], [4], [5].

The modulating effect of valence on the early stage of affective processing has been attributed to the biological significance of negative stimuli [2], [6]. At the early stage, several ERP components (e. g., P1, N1, P2) elicited by negative stimuli have been found to be more pronounced than those elicited by positive and neutral stimuli [7], [8], [9], [10]. However, such valence effects are not consistent among previous studies. For instance, comparable early ERP components elicited by negative and positive stimuli have also been reported [5], [11], [12], [13], [14]. Additionally, a considerable number of studies have even reported that early ERP components elicited by positive stimuli can be more pronounced than those elicited by negative stimuli, possibly due to the greater “automatic arousal” of positive stimuli [12], [15], [16], [17].

In contrast, an association between arousal effects and late ERP components is prevalent in previous studies; in particular, arousal effects usually occurred at around 250 ms and were accentuated over the following several seconds [15], [18], [19]. For instance, high arousing (negative and positive) stimuli have been found to elicit more pronounced late ERP components (e. g., N2, P3) than low arousing (neutral) stimuli, even when the affective stimuli were briefly presented [5], [12], [14], [15], [20], [21]. The modulating effects of arousal on late ERP components were suggested to reflect motivation-driven attention to high arousing stimuli, indicating that meaningful stimuli are selectively processed at the late stage [22]. However, such arousal effects have not been consistently observed, and valence effects on late ERP components have also been reported [23], [24], [25]. Current evidence is thus unclear as to whether valence, arousal, or both factors together modulate the late processing of affective stimuli [18].

Although valence and arousal have been suggested to modulate ERP components at relatively separate stages [2], recent behavioral and fMRI studies have found that the effects of arousal and valence are more complicated than the ERP studies have suggested, especially in their interactions [17], [26], [27], [28], [29], [30], [31]. For example, in a behavioral study, Robinson et al. found that participants responded faster to high arousing negative and low arousing positive stimuli than to low arousing negative and high arousing positive stimuli. Subsequent studies further demonstrated that the interaction between valence and arousal can be elicited automatically [32]. Such an interaction has been further confirmed in recent fMRI studies. In a study assessing the neural responses to affective items which are successfully recognized, Steinmetz and his colleagues found that high arousing negative and low arousing positive stimuli enhanced connections between the amygdala and other regions, whereas low arousing negative and high arousing positive stimuli reduced these connections [30]. One way to interpret the valence by arousal interaction is using the model of evaluative space proposed by Cacioppo and his colleagues [33], [34], [35], [36], [37], [38]. According to this model, motivational systems for positive and negative valence processing (appetitive system and aversive system, respectively) are separable and are characterized by different activation functions [39]: whereas the appetitive motivational system tends to respond more than the aversive motivational system when the evaluative input (as indicated by arousal ratings) is low (positive offset), the aversive motivational system responds more than appetitive motivational system when the evaluative input is high [36], [38]. This occurs because the aversive motivational system tends to response more intensely than appetitive motivational to comparable increases in input (negative bias). The positive offset and negative bias could well explain the valence by arousal interaction found in behavioral and fMRI studies [29], [30], [32], [40], as well as the inconsistent valence effects found in previous ERP studies.

Whereas interactions between valence and arousal have been amply demonstrated in behavioral and fMRI studies, there is little evidence for such interactions from ERP studies [2], [17]. A number of ERP studies have been conducted with orthogonal factors of valence by arousal [4], [5], [17], [18], [20], [41], but only one of these revealed a significant interaction between valence and arousal, which was found by analyzing ERP topographies rather than ERP components [4].

Previous ERP studies investigating affective processing often used explicit tasks such as the passive viewing task, oddball paradigm, and affective categorization task [4], [17], [18], [19], [23], [24]. However, numerous studies have shown that affective content can be processed even when it is not explicitly attended [42], [43], [44], [45], [46]. For example, when participants are instructed to categorize affective pictures according to nonaffective features of these stimuli (e.g., identifying how many people in the pictures), affective information has been found to modulate ERP components at both early and late stages [7], [8], [10], [45], [47], [48]. Nevertheless, how such implicit affective processing can be influenced by valence and arousal and what are the corresponding ERP correlates are yet under debate [8], [43], [48]. For instance, previous studies have frequently associated P300 with arousal in implicit affective processing [47], [48], [49], but Carretie and his colleagues [43], [44] demonstrated that the valence-associated N300 (275–325 ms) might be a better index than P300 to reflect implicit processing of affective stimuli.

In sum, the exact contributions of valence and arousal in affective processing are unclear in previous studies, and an orthogonal design of valence by arousal would provide a promising means to systematically investigate their independent and interactive effects on affective processing.

In the present study, we aimed to investigate the electrophysiological correlates of the implicit processing of affective stimuli. Compared to previous implicit tasks in which participants categorized affective stimuli according to nonaffective features of pictures [7], [8], [43], [46], [47], [49], [50], [51], our task was even more “implicit” in that participants did not even need to attend to and process the content of the pictures but merely needed to judge the color of the picture frame [10], [52]. In addition, we employed an orthogonal design of valence by arousal so that both the main effects and interaction could be systematically investigated. According to Cacioppo et al.'s model and prior behavioral findings [29], [32], both faster RTs and higher amplitude ERPs might reflect enhanced responses to the appetitive or aversive motivational system [53], [54]. Accordingly, we hypothesized that when the arousal ratings of stimuli were low, positive stimuli would elicit larger ERPs than negative stimuli, whereas when arousal ratings were high, positive stimuli would elicit smaller ERPs than negative stimuli.

## Methods

### Participants

Twenty-one individuals (10 females) aged 18–29 years (mean age = 21 years) participated in the present study for payment. All participants had normal or corrected-to-normal vision and did not have any history of psychiatric or neurological illness. This study and the recruitment of participants were approved by the Beijing Normal University Institutional Review Board (IRB). Written informed consents were collected for all participants.

### Stimuli

Pictures used in our study were selected from the International Affective Picture System based on normative ratings of valence and arousal [3]. There were four stimuli types: high arousing positive (HP), low arousing positive (LP), high arousing negative (HN), and low arousing negative (LN), with 15 pictures for each type (HP: 2216, 4607, 4658, 4687, 5470, 5621, 5629, 8030, 8034, 8090, 8180, 8190, 8370, 8470, 8490; LP: 1500, 1590, 1670, 2080, 2304, 2340, 2360, 2500, 2515, 5220, 7238, 7285, 7430, 7545, 8320; HN: 3053, 3060, 3071, 3102, 3120, 3130, 3170, 3261, 3350, 3400, 3530, 6313, 6570, 9570, 9921; LN: 2205, 2700, 2722, 2750, 2753, 9001, 9041, 9046, 9265, 9280, 9320, 6010, 9330, 9415, 9830). The valence and arousal ratings of these pictures are listed in Table 1. Positive and negative pictures were significantly different in valence ratings (*p*<.001), but not in arousal ratings (*p* = .41); whereas high arousing and low arousing pictures were significantly different in arousal ratings (*p*<.001), but not in valence ratings (*p* = .55). All pictures were adjusted to a size of 12°×9° and were surrounded on a random basis by one of four colored frames (red, yellow, blue, or green) having a width of 0.5°. Pictures were presented on a CRT monitor with an 80 Hz refresh rate using the Psychophysics Toolbox extensions for Matlab [55], [56].

**Table 1:** The mean (with SD) valence and arousal ratings , reaction times (RT, ms) and accuracy (%) in each condition.

	Positive		Negative	
	High arousal	Low arousal	High arousal	Low arousal
Valence	7.29(.39)	6.93(.71)	1.78(.29)	2.92(.44)
Arousal	6.43(.43)	4.03(.56)	6.64(.47)	4.38(.42)
RTs	816.68(123.72)	825.90(102.81)	836.39(159.31)	796.97(119.63)
Accuracy	96.95(3.51)	97.13(2.17)	96.06(4.61)	96.05(3.83)

### Procedure

**Figure 1:**
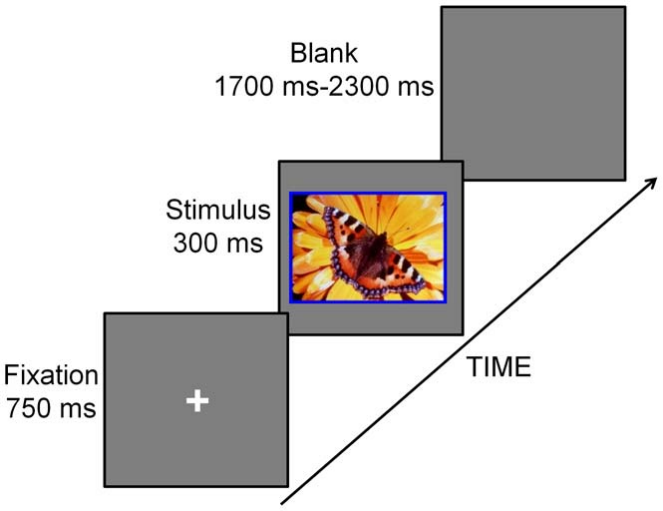
Experimental procedure. A fixation point was first presented for 750 ms. Then an affective picture with a colored frame (either red, yellow, blue, or green) was presented for 300 ms. The participant was instructed to response to the color of the frame as quickly and accurately as possible by pressing one of four separate keys. The inter-trial interval was randomized between 1700 and 2300 ms.

The experimental procedure is shown in Figure 1, each trial began with a fixation (750 ms) point, followed by the stimulus for 300 ms. The inter-trial interval was randomized between 1700 and 2300 ms. Participants were instructed to judge the color of the frame as quickly and accurately as possible by pressing one of four corresponding keys. The whole experiment consisted of four blocks of each of the four stimulus types [13], [57], [58], [59]. Each block contained 75 trials. The orders of four blocks were randomized across participants, and the sequence of trials in each block was pseudo-random, with the constraints that identical pictures were not presented successively and picture frames with the same color were not presented successively more than three times. Participants sat in a dimly lit and sound-attenuated chamber facing a CRT monitor 80 cm away from their eyes. Before the formal experiment, each participant was given a training block to get familiar with response keys. Four pictures that were not included in the formal experiment were used in a 20 trial of training block.

### Electrophysiological recording

The electroencephalogram (EEG) was recorded from 64 scalp sites using electrodes mounted in an elastic cap (Brain Product, GmbH, Germany), with an online reference to the left mastoid. The horizontal electroencephalogram (HEOG) was recorded with two electrodes placing laterally to the right and left eyes. The vertical electroencephalogram (VEOG) was recorded with electrodes placed above and below the right eye. All inter-electrode impedances were maintained below 10 kΩ. The EEG and EOG were amplified using a 0.01–100 Hz band-pass and continuously sampled at 500 Hz in each channel for off-line analysis. All EEG signals were re-referenced off-line to the average of left and right mastoids [60]. The EEG data were low-pass filtered below 30 Hz (24 dB/oct) and were corrected for eye movements or blinks using the Gratton and Coles [61] method as implemented in the Brain Vision analysis software (Brain Product, GmbH, Germany). Trials containing EEG sweeps with amplitudes exceeding ±100 µV were excluded.

### Data reduction and analysis

**Figure 2:**
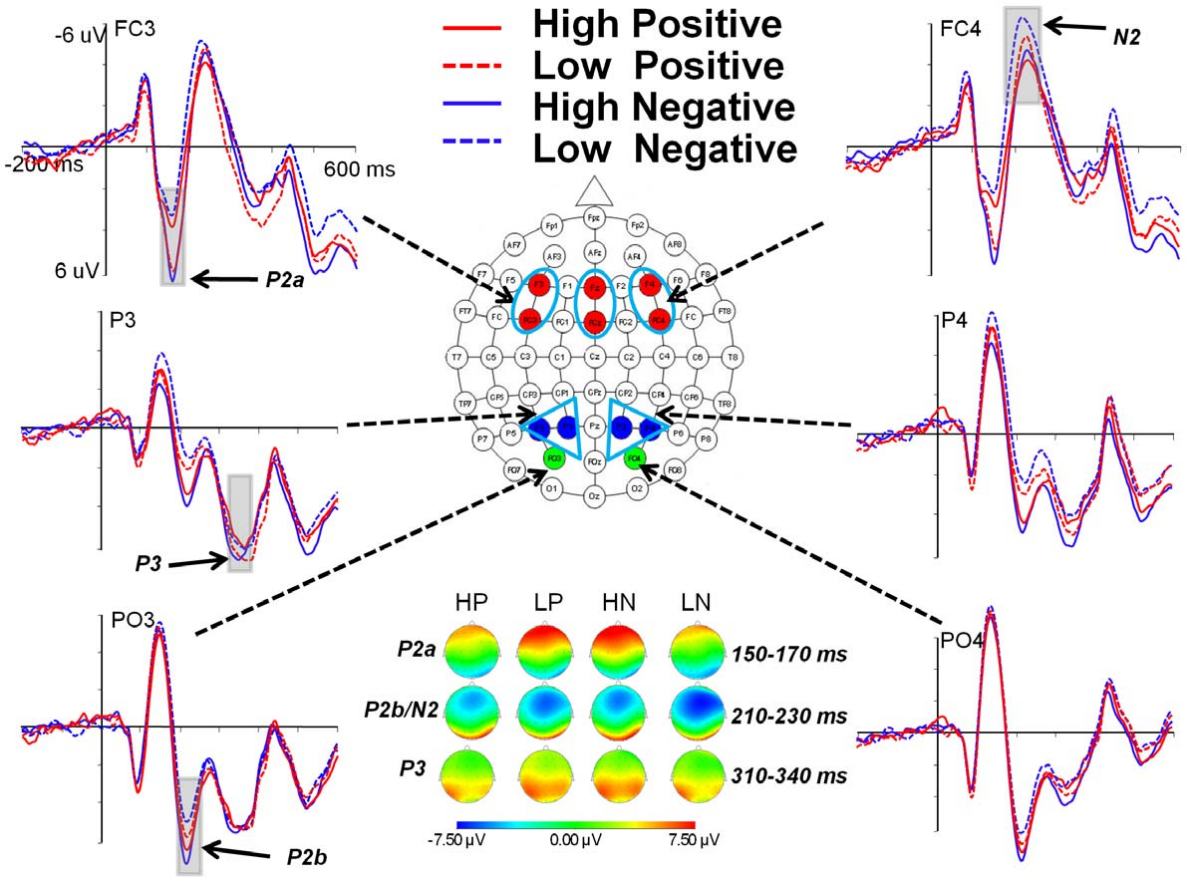
The grand average ERPs evoked by high arousing positive (HP), low arousing positive (LP), high arousing negative (HN), and low arousing negative (LN) pictures. Compared to HP and LN, HN and LP stimuli elicited both larger P2a and P3 components at bilateral frontal (F3, F4, FC3, and FC4) and parietal regions (P1, P2, P3 and P4), respectively. Compared to Low arousing pictures, high arousing pictures elicited a larger P2b component at posterior parieto-occipital regions (PO3 and PO4). Furthermore, the arousal effects on the N2 component were restricted to the negative pictures. The voltage topographies for P2a (150–170 ms), P2b/N2 (210–230 ms), and P3 (310–340 ms) components in each condition are shown.

ERPs elicited by each type of stimulus were averaged separately over an epoch of 800 ms with a 200 ms pre-stimulus baseline. Based on inspection of the grand-averaged ERP waveforms (Figure 2) and previous studies of affective picture processing [7], [13], [14], [22], four different ERP components, P2a, P2b, N2, and P3 were analyzed in the current study. Different sets of electrodes in the frontal and parietal regions were chosen for the baseline-to-peak measurements of these components: FC3, FCz, FC4, F3, Fz, and F4 for P2a (100–200 ms) and N2 (200–300 ms) components; PO3 and PO4 for the P2b (200–300 ms) component; P1, P3, P2 and P4 for the P3 (300–400 ms) component. The amplitudes and latencies of these components were then analyzed using repeated measures ANOVAs with the factors of Valence (negative *vs.* positive) by Arousal (low *vs.* high) by Hemisphere (left *vs.* right) by Electrodes (electrodes selected for particular ERP component, as stated above). *P* values were adjusted according to the Greenhouse-Geisser correction if necessary. Bonferroni correction was used for multiple comparisons.

## Results

### Behavioral results

The behavioral results are shown in Table 1. Two-way repeated measures ANOVAs of Valence (negative *vs.* positive) by Arousal (low *vs.* high) on reaction times (RTs) and accuracy revealed neither significant main effects of Valence (RTs: *F*(1, 20) = .10, *p* = .75; accuracy: *F*(1, 20) = 2.26, *p* = .15) nor Arousal (RTs: *F*(1, 20) = .74, *p* = .40; accuracy: *F*(1, 20) = .02, *p* = .90), nor significant interactions of Valence by Arousal, (RTs: *F*(1, 20) = 2.27, *p* = .15; accuracy: *F*(1, 20) = .03, *p* = .86). Thus, behaviorally participants responded similarly to the frames of affective pictures having different valence and arousal values, indicating that this implicit viewing task required no effortful processing of the affective stimuli [13], [59].

### ERP results

#### P2a (100–200 ms)

**Figure 3:**
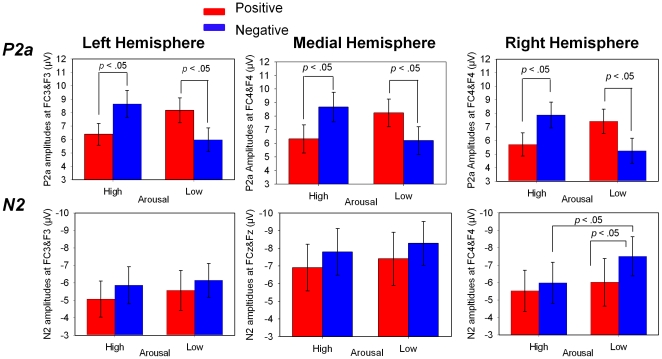
The amplitudes of P2a and N2 evoked by high arousing positive (HP), low arousing positive (LP), high arousing negative (HN), and low arousing negative (LN) pictures (error bars show 1 SE). Interaction between Valence and Arousal was found on P2a and N2. Specifically, valence-arousal-congruent pictures (HN and LP) elicited larger P2a than valence-arousal-incongruent pictures (LN and HP). In addition, the arousal effects for N2were restricted to negative pictures, showing that LN elicited larger N2 than HN. Moreover, the interaction between Valence and Arousal on N2 was restricted to the right hemisphere.

Four-way repeated measures ANOVA of Valence (negative *vs.* positive) by Arousal (low *vs.* high) by Hemisphere (left *vs.* medial *vs.* right) by Electrode (F3, Fz and F4 *vs.* FC3, FCz, and FC4) on P2a amplitudes yielded a significant main effect of Hemisphere (F(2, 40) = 2.25, p = .049,*η^2^_p_* = .14) and Electrode (*F*(1, 20) = 27.04, *p*<.001,*η^2^_p_* = .58), as well as a significant interaction of and Hemisphere by Electrode (F(2, 40) = 3.79, p = .031,*η^2^_p_* = .16) and Valence by Arousal (*F*(1, 20) = 19.29, *p*<.001,*η^2^_p_* = .49), such that P2a amplitudes elicited by high arousing negative pictures were larger than those by high arousing positive pictures; whereas the P2a amplitudes elicited by low arousing negative pictures were smaller than those by lowing arousing positive pictures (Figures 2 and 3, Table 2). Such a Valence by Arousal interaction has rarely been reported in previous ERP studies in which the valence effect was related to this early stage [2], [51]. Further analysis also revealed that the P2a amplitudes were larger at F3, Fz, and F4 (*M* = 7.82 µV, *SD* = 4.19) than at FC3, FCz, and FC4 (*M* = 6.33 µV, *SD* = 3.69). Four-way repeated measures ANOVA of Valence by Arousal by Hemisphere by Electrode on P2a latencies (Table 3) yielded no significant effects.

**Table 2:** The mean (with SD) peak amplitude (µV) of each component in response to different affective pictures.

	Positive		Negative	
	High arousal	Low arousal	High arousal	Low arousal
P2a	6.14(4.03)	7.95(4.23)	8.40(4.56)	5.81(4.23)
N2	−5.84(5.31)	−6.33(6.04)	−6.55(5.32)	−7.31(5.02)
P2b	7.97(4.00)	7.35(4.01)	8.42(4.81)	6.65(3.59)
P3	6.12(5.25)	7.00(5.09)	7.28(4.87)	5.99(4.83)

**Table 3:** The mean (with SD) peak latency (ms) of each component in response to different affective pictures.

	Positive		Negative	
	High arousal	Low arousal	High arousal	Low arousal
P2a	151.46(20.01)	151.48(17.10)	152.25(15.69)	147.56(18.16)
N2	242.06(18.93)	238.27(18.38)	240.29(19.43)	233.37(22.78)
P2b	226.05(20.90)	228.95(25.55)	222.24(17.15)	231.62(28.61)
P3	349.69(19.65)	353.95(26.83)	345.19(20.94)	344.40(23.85)

#### N2(200–300 ms)

Four-way repeated measures ANOVA of Valence (negative *vs.* positive) by Arousal (low *vs.* high) by Hemisphere (left *vs.* medial *vs.* right) by Electrode (F3, Fz, and F4 *vs.* FC3, FCz, and FC4) on N2 amplitudes yielded significant main effects of Valence (*F*(1, 20) = 5.71, *p* = .027,*η^2^_p_* = .22) and Hemisphere (*F*(2, 40) = 12.94, *p*<.001,*η^2^_p_* = .39), two-way interactions of Arousal by Hemisphere (*F*(2, 40) = 4.33, *p* = .021,*η^2^_p_* = .18), Arousal by Electrode (*F*(1, 20) = 8.37, *p* = .009,*η^2^_p_* = .30) and Hemisphere by Electrode (*F*(2, 40) = 6.52, *p* = .006,*η^2^_p_* = .25), as well as a three-way interaction of Valence by Arousal by Hemisphere (*F*(2, 40) = 6.08, *p* = .006,*η^2^_p_* = .23). Further analysis revealed that over the right hemisphere, N2 amplitudes were more negative for low arousing negative pictures than for high arousing negative pictures; whereas N2 amplitudes elicited by high and low arousing positive pictures showed no significant difference. In addition, N2 amplitudes were more negative for low arousing negative pictures than for low arousing positive pictures; whereas N2 amplitudes elicited by high arousing negative and positive pictures showed no significant difference (Figure 2 and 3, Table 2). In contrast, over the left and medial sites, no effects were found on the N2 amplitudes. These results indicated that the arousal effects on N2 depended on valence, and such an interaction was restricted to the right hemisphere. Furthermore, N2 amplitudes at medial sites (*M* = −7.60 µV, *SD* = 5.94) were more negative than at right (*M* = −6.26 µV, *SD* = 5.37) and left (*M* = −5.66 µV, *SD* = 4.57) sites, which were consistent with previous findings [10], [62]. Four-way repeated measures ANOVA of Valence by Arousal by Hemisphere by Electrode on N2 latencies (Table 3) only revealed a significant interaction of Hemisphere by Electrode, *F*(2, 40) = 4.81, *p* = .014,*η^2^_p_* = .19. Further analysis revealed that N2 latencies were shorter at FCz (*M* = 238.55 ms, *SD* = 17.68) than at Fz (*M* = 241.02 ms, *SD* = 19.08).

#### P2b (200–300 ms)

**Figure 4:**
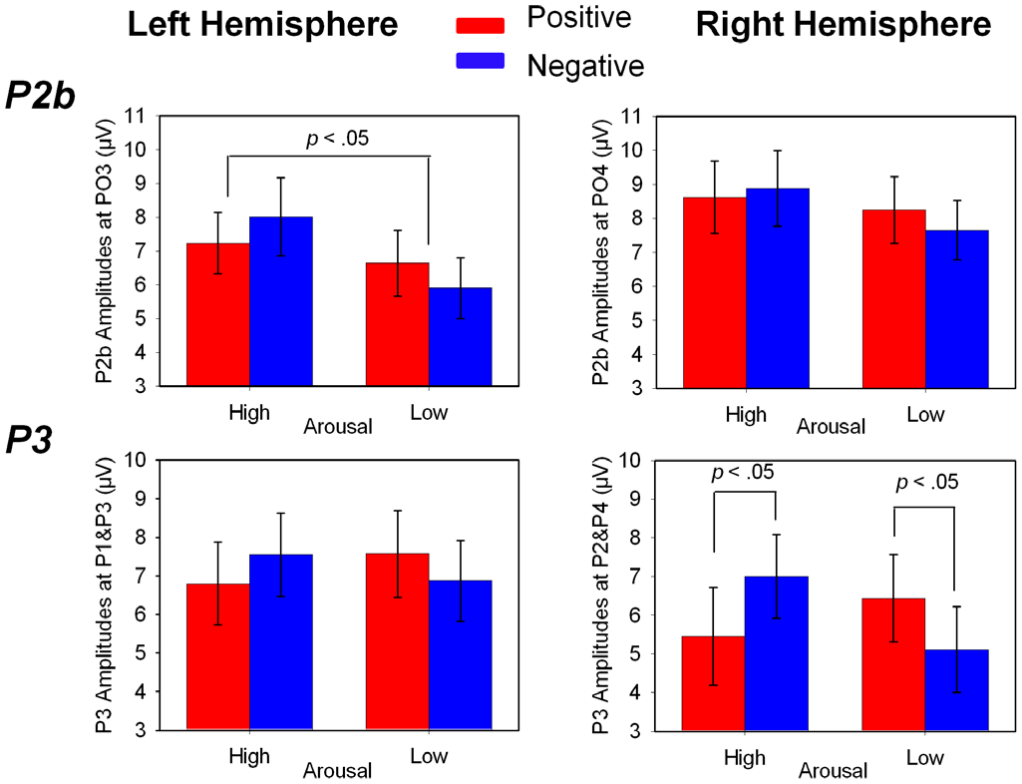
The amplitudes of P2b and P3 evoked by high arousing positive (HP), low arousing positive (LP), high arousing negative (HN), and low arousing negative (LN) pictures (error bars show 1 SE). The main effect of Arousal was only found on P2b, such that high arousing pictures elicited larger P2b than low arousing pictures. Interaction between Valence and Arousal was found on P3. Specifically, valence-arousal-congruent pictures (HN and LP) elicited larger P3 than valence-arousal-incongruent pictures (LN and HP). In addition, the interaction between Valence and Arousal on P3 was restricted to the right hemisphere.

Three-way repeated measures ANOVA of Valence (negative *vs.* positive) by Arousal (low *vs.* high) by Hemisphere (left: PO3 *vs.* right: PO4) on P2b amplitudes yielded only a significant main effect of Arousal, *F* (1, 20) = 4.93, *p* = .038,*η^2^_p_* = .20, such that high arousing pictures elicited larger P2b than low arousing pictures (Figure 2 and 4, Table 2). Neither the main effect of Valence (*F*(1, 20) = .13, *p* = .72) nor the interaction between Valence and Arousal (*F*(1, 20) = 3.74, *p* = .067) was significant. These results indicated that the arousal information of affective stimuli could be independently processed within this stage [14], [22]. Three-way repeated measures ANOVA of Valence by Arousal by Hemisphere on P2b latencies (Table 3) revealed a marginally significant main effect of Arousal (*F*(1, 20) = 4.50, *p* = .047,*η^2^_p_* = .18), and a three-way interaction of Valence, Arousal, and Hemisphere (*F*(1, 20) = 4.45, *p* = .048,*η^2^_p_* = .18), such that the latencies for high arousing negative pictures were shorter than those for low arousing negative pictures over the left hemisphere. These results were consistent with previous studies showing that P2b latencies were shorter for low arousing stimuli than for high arousing stimuli [63].

#### P3(300–400 ms)

Four-way repeated measures ANOVA of Valence (negative *vs.* positive) by Arousal (low *vs.* high) by Hemisphere (left *vs.* right) by Electrode (P1 and P2 *vs.* P3 and P4) on P3 amplitudes yielded a significant main effect of Hemisphere (*F*(1, 20) = 11.80, *p* = .003,*η^2^_p_* = .37), a two-way interaction of Valence by Arousal (*F*(1, 20) = 8.52, *p* = .008,*η^2^_p_* = .30), as well as a three-way interaction of Valence by Arousal by Hemisphere (*F*(1, 20) = 9.20, *p* = .007,*η^2^_p_* = .32). Further analysis revealed that the P3 amplitudes were larger over the left hemisphere than over the right hemisphere. Interestingly, P3 showed a Valence by Arousal interaction similar to P2a over the right hemisphere, such that the P3 amplitudes for high arousing negative pictures were larger than for high arousing positive pictures; whereas P3 amplitudes elicited by low arousing negative pictures were smaller than those by low arousing positive pictures (Figure 2 and 4, Table 2). Such an interaction was not found over the left hemisphere. Such a Valence by Arousal interaction pattern on ERP components has rarely been previously reported, although the interaction of valence and arousal on affective processing at this stage has been suggested [4], [41]. Four-way repeated measures ANOVA of Valence by Arousal by Hemisphere by Electrode on P3 latencies (Table 3) revealed significant main effect of Valence (*F*(1, 20) = 6.17, *p* = .022,*η^2^_p_* = .24) and Electrode (*F*(1, 20) = 12.36, *p* = .002,*η^2^_p_* = .38), such that the latencies of negative pictures were shorter than those of positive pictures; and P3 latencies at P1 and P2 electrodes were shorter than those at P3 and P4 electrodes. Such results of P3 latencies are consistent with previous studies showing that latency reductions at posterior sites were restricted to negative stimuli [64].

## Discussion

In the present study, we investigated the influence of valence and arousal on the implicit processing of affective pictures using the ERP technique. We found significant interactions between valence and arousal within both early and late time windows over both parietal and frontal sites, which were reflected in three different ERP components: P2a (100–200 ms), N2 (200–300 ms), and P3 (300–400 ms). In addition, we also found a significant main effect of arousal on the P2b (200–300 ms) component over parieto-occipital sites. These results indicate that valence and arousal play interactive roles in the implicit processing of affective stimuli.

An interaction between valence and arousal effects was found on both the early P2a and late P3 components, such that high arousing negative (HN) and low arousing positive (LP) pictures elicited larger P2a and P3 amplitudes than high arousing positive (HP) and low arousing negative (LN) pictures. These results are consistent with previous behavioral results showing that participants responded faster to HN and LP stimuli than to HP and LN stimuli [29]. Robinson and his colleagues [29] proposed that because high arousing stimuli, no matter whether positive or negative, are negatively coded, the HN and LP stimuli are thus congruent, whereas HP and LN stimuli are incongruent in the valence and arousal dimensions. The observed RT differences could be explained by assuming that valence-arousal-incongruent stimuli (HP and LN) are evaluated more slowly than valence-arousal-congruent stimuli (HN and LP). In another study with an affective Simon task, Eder and Rothermund further demonstrated that the congruency of valence and arousal dimensions can affect processings of stimuli even when participants do not attend to their affective contents. They thus concluded that arousal interacts with valence in stimulus processing, and such interactions occur preattentively [32]. In agreement with these behavioral studies, a recent ERP study found that the interaction between valence and arousal can occur within the interval 302–330 ms [4]. Using the ERP microstate analysis of topography, Gianocci and colleagues found an interesting “common step” between the processing of valence and arousal, such that the last microstate of valence extraction was identical to the first microstate of arousal extraction. This microstate lasted from 302 to 330 ms after the stimulus onset. The authors suggested that such a “common step” reflects the interaction between valence and arousal on affective processing [4].

Our results support and extend previous behavioral findings showing reaction time differences between HN & LP and HP & LN stimuli [29]. Our ERP results indicate that these behavioral differences might be associated with the P2a and P3 components. These results suggest that HN & LP stimuli evoke more affective processing at both early and late stages than HP & LN stimuli.

One possible theoretical explanation for our finding is based on the model of evaluative space proposed by Cacioppo et al. [33], [34]. According to this model, stimuli of low arousal will generate a positive offset that suggests a larger response to positive stimuli (positive offset), whereas stimuli of high arousal will generate an enhanced response to negative stimuli (negativity bias). Both positive offset and negativity bias have been observed in previous behavioral studies [29], [32] (but see [65] for exception), but only negativity bias has been emphasized in ERP studies [9], [66]; such valence effects in ERPs have been considered inconsistent, however [2]. Our results supported and extended previous behavioral findings by showing that positive offset and negativity bias effects can occur at both early (P2a) and later (P3) stages of processing.

In addition, we found a significant interaction between valence and arousal on the N2 component, but with a different pattern than P2a and P3, such that N2 amplitudes were larger for LN than LP, but were identical for HN and HP (Fig.3). Moreover, such an interaction was restricted to the right hemisphere. In previous studies, N2 has been considered as a complex component comprised of several sub-components, some reflecting automatic affective processing and others reflecting executive control processing [10]. The N2 effect found in the current study might be related to both of these. On the one hand, previous studies showed that the arousal effect can automatically modulate ERP components within 200–300 ms, even when the cognitive resource is limited [2], [14], [67]. For instance, compared to high arousing negative and positive pictures, low arousing neutral pictures elicit a negative going component within 200–300 ms at frontal sites [68], [69], even when pictures are briefly presented [14], [22]. Olofsson et al. proposed that the arousal effect between 200–300 ms is related to the amygdala's response to affective stimuli. In our study, we found that low arousing pictures elicited a more negative N2 than high arousing pictures; such arousal effects, however, were only found for negative stimuli [69]. These results suggest that the interaction between valence and arousal on the amygdala might occur within 200–300 ms. On the other hand, the N2 effects found in our study might reflect the valence-related influence on affective processing that has been attributed to the exertion of cognitive control on threat-related stimuli [10], [70], [71]. Consistent with this view, a recent fMRI study found that negative pictures elicit stronger dorsolateral prefrontal activation than positive pictures when participants perform an implicit task [70]. Additionally, the N2 effects we found were prevalent over the right hemisphere, which accords with previous studies showing that negative affective processing is primarily a function of the right hemisphere [67], . In either case, our study demonstrated that valence and arousal also played an interactive role in implicit affective processing at this stage.

In addition to the above-described interactions between valence and arousal, we also found a significant main effect of arousal on the posterior P2b component, such that high arousing pictures elicited larger P2b than low arousing pictures. Such an arousal effect has also been reported in previous studies [50], [75].

In reviewing ERP studies of affective processing in recent decades, Olofsson et al. suggested that valence modulates early ERP components and arousal modulates late ERP components [69]. The present results with implicit affective processing suggest that their proposal might be oversimplified. Two possible reasons might account for why our results were different from previous findings in emotion evaluation tasks. Firstly, participants in previous emotion evaluation tasks were asked to categorize emotional pictures, or simply to look at the emotional pictures. Such manipulations are likely to lead participants to assume that several kinds of affective stimuli (e.g., negative, high arousing stimuli) are more relevant to the task than others [7], [43], [44], [75]. Such a “relevance-for-task effect” has been found to modulate ERP components [44]. Thus, implicit tasks (such as the one used in the present study) might provide a better way to study the valence and arousal effects related to affective processing, because they could avoid the interference of the “relevance-for-task effect”. Secondly, according to the model of evaluation space proposed by Cacioppo [33], [76], when the evaluative activation is low, the intrinsic response of an individual to affective stimuli is that the motivation to explore is stronger than the motivation to avoid (positive offset). However, in previous emotion evaluation tasks (especially in the categorization task), participants were instructed to categorize affective pictures according to their valence, which means they were probably motivated to explore every presented picture. Such a manipulation might cause “task-related motivation”, which would influence the individual's intrinsic response to affective pictures. In contrast, participants in implicit tasks are asked to do tasks that are unrelated to the affective picture. Such a manipulation would avoid interference from “task-related motivation”; therefore the individual's intrinsic response to affective stimuli would be more likely to be observed in implicit tasks. In summary, using implicit tasks could avoid the “relevance-for-task effect” and “task-related motivation” that might interfere with an individual's intrinsic response to affective stimuli; this might explain why the results observed in the present study were different from prior findings with emotion evaluation tasks.

A limitation of the present study is that we did not screen participants in terms of possible anxiety or depressive states. Although previous behavioral and fMRI studies have observed that anxiety and depressive states modulate the processing of affective pictures [77], [78], [79], [80], [81], such influence has been frequently ignored in ERP studies [2], [17], [48], [51]. Several recent studies have indicated that ERPs elicited by affective pictures can be modulated by anxiety states [82], [83], [84], suggesting a promising approach to characterize clinical affective dysfunction [2]. Thus, the modulations of anxiety and depressive states on ERPs evoked by affective pictures deserve systematic investigation in future studies. Another limitation of the current study is that we did not add a blank screen between fixation and picture (see Figure 1) to avoid possible contamination of the ERPs evoked by pictures by a fixation offset effect. Although such an offset effect is generally weak [85], and similar stimulus sequences have been used in previous studies [82], [84], this experimental design flaw should be avoided in future studies.
